# High sensitivity of an ELISA kit for detection of the gamma-isoform of 14-3-3 proteins: usefulness in laboratory diagnosis of human prion disease

**DOI:** 10.1186/1471-2377-11-120

**Published:** 2011-10-04

**Authors:** Yuki Matsui, Katsuya Satoh, Toshiaki Miyazaki, Susumu Shirabe, Ryuichiro Atarashi, Kazuo Mutsukura, Akira Satoh, Yasufumi Kataoka, Noriyuki Nishida

**Affiliations:** 1 Department of Pharmaceutical Care and Health Sciences, Faculty of Pharmaceutical Sciences, Fukuoka University, 8-19-1 Nanakuma, Fukuoka 814-0180, Japan; 2 Department of Molecular Microbiology and Immunology, Graduate School of Biomedical Science, Nagasaki University, 1-12-4 Sakamoto, Nagasaki 852-8523, Japan; 3 Cyclex Co.LTD, 1063-103 Ohara, Terasawaoka, Ina, Nagano 396-0002, Japan; 4 Center for Community and Campus Health, Nagasaki University, 1-14 Bunkyo, Nagasaki 852-8521, Japan; 5 Nagasaki Kita Hospital, 800 Tokitsu, Nagasaki 851-2103, Japan

**Keywords:** CJD, CSF, ELISA, prion disease, 14-3-3 protein, tau protein

## Abstract

**Background:**

The gamma-isoform of the 14-3-3 protein (14-3-3 gamma) is expressed in neurons, and could be a specific marker for neuronal damage. This protein has been reported as a detectable biomarker, especially in the cerebrospinal fluid (CSF) of Creutzfeldt-Jakob disease (CJD) patients by Western blotting (WB) or enzyme-linked immunosorbent assays (ELISAs). Western blotting for 14-3-3 gamma is not sensitive, and the reported data are conflicting among publications. An ELISA specific for 14-3-3 gamma is not available.

**Methods:**

CJD patients (n = 114 sporadic CJD patients, 7 genetic CJD, and 3 iatrogenic CJD) and 99 patients with other neurodegenerative diseases were examined in this study. The CSF samples obtained were analyzed by Western blotting for 14-3-3 gamma, and by ELISA for total tau protein. We evaluated the sensitivity and specificity of the newly developed sandwich ELISA for 14-3-3 gamma.

**Results:**

The cut-off value of the 14-3-3 gamma ELISA was > 1, 683 AU/ml; and sensitivity was 95.2%, with 72.7% specificity. This specificity was the same for the total tau protein ELISA. Seven CJD cases were negative by WB but positive using the 14-3-3 gamma ELISA, indicating that the ELISA is more sensitive. All 21 cases of early stage CJD could be diagnosed using a combination of the 14-3-3γ ELISA and diffusion weighted MR imaging (DWI-MRI).

**Conclusion:**

The 14-3-3 gamma ELISA was more sensitive than conventional WB, and was useful for laboratory diagnosis of CJD, similar to the ELISA for the tau protein. Using DWI-MRI and these ELISA tests on CSF, diagnosis of CJD will be possible even at early stages of the disease.

## Background

Hshich et al.[1] reported use of the 14-3-3 protein for diagnosis of prion diseases in 1996. This protein is a reliable marker of rapid neuronal destruction, and has been detected in the cerebrospinal fluid (CSF) of several progressive neurological disorders. The 14-3-3 protein is one supportive and essential marker in the CSF of sporadic Creutzfeldt-Jakob disease (CJD) patients. Periodic sharp wave complexes (PSWC) observed on an electroencephalographic (EEG) recording and the presence of 14-3-3 in CSF are both included in the diagnostic criteria for CJD as supplied by the World Health Organization (WHO) [2]. The 14-3-3 protein is detected by Western blot (WB) in many clinical laboratories; however, conducting a WB assay to detect 14-3-3 is time consuming and expensive because the WB method consists of many steps and requires multiple investigators to discern the protein bands [3]. Thus, the development of a standard 14-3-3 protein assay and a valid criterion for quantitative assessment is urgently required.

The 14-3-3 protein has been reported to be a detectable biomarker, especially in the CSF of CJD patients, by WB or enzyme-linked immunosorbent assay (ELISA). However, the WB to detect 14-3-3 is not very sensitive enough and the data obtained to date differ among reports.

Previous studies have utilized an ELISA to detect 14-3-3 in the CSF [4,5] (Additional file 1, table S1), but four problems were encountered: the number of CJD patients was less than 50; the make-up of the CJD and case-control groups was not clear; the analytic means of the cut-off data for the ELISA were unclear; and the specificity was very low. We attempted to improve upon these four problems and attempted to develop a new, specific, 14-3-3γ ELISA.

We developed a specific ELISA using the gamma isoform of 14-3-3 (14-3-3γ). Additionally, results from the WB and ELISA analyses were compared. We analyzed 124 human prion disease cases and 99 human non-prion disease cases using the 14-3-3γ ELISA and assessed if quantification of 14-3-3 might be helpful in their differentiation.

## Methods

### 1. Patients

Patients with suspected CJD were recruited from hospitals all over Japan for the purpose of conducting biochemical CSF assays. From more than 300 requests, a follow-up study was performed with 124 CJD patients. The cases were classified as sporadic CJD (n = 114), genetic CJD (n = 7; four cases with a V180I mutation, two cases with an M232R mutation and a single case with an E200K mutation in the prion protein gene), or iatrogenic CJD (dura-associated CJD; n = 3). A total of 114 sporadic CJD patients and 3 iatrogenic CJD patients did not express a *prion protein gene *(*PRNP*) mutation. All 124 cases contained MM at codon 129 and EE at codon 219 of the *PRNP*.

Subjects for the present study consisted of 114 sporadic CJD patients with confirmed CJD diagnosis. All 114 sporadic patients fulfilled the WHO diagnostic criteria for CJD. All sporadic CJD cases were typical with respect to clinical findings, clinical time course, neuroimaging [FLAIR and diffusion-weighted magnetic resonance imaging (DWI-MRI) with high signal abnormalities in the caudate nucleus and putamen, or at least two cortical regions] and PSWCs via EEG.

A total of seven sporadic CJD cases (four males, three females) were definite, and 107 sporadic cases were probable among the 114 sporadic CJD patients. Two cases (V180I; females) were definite cases among the seven genetic CJD cases, and one case was a definite among the three iatrogenic CJD cases.

For control samples, CSF was collected from 99 patients who suffered from one of the following disorders: dementia of Alzheimer's type (DAT; n = 54, 33 male, 21 female), cerebrovascular dementia (n = 7, 5 male, 2 female), Parkinson's disease (n = 5, 4 male, 1 female), progressive supranuclear palsy (PSP; n = 3, 2 male, 1 female), frontotemporal lobular degeneration (n = 2, 1 male, 1 female), Huntington's disease (n = 1; 1 male), corticobasal degeneration (n = 2, both female), amyotrophic lateral sclerosis (n = 3, 1 male, 2 female), limbic encephalitis (n = 2, 1 male, 1 female), mitochondrial myopathy, encephalopathy, lactic acidosis and stroke-like episodes (MELAS; n = 4, 2 male, 2 female), paraneoplastic cerebellar disorder/Lambert-Eaton myasthenic syndrome (PCD/LEMS; n = 2, 1 male, 1 female), temporal epilepsy (n = 4, 1 male, 3 female), mild cognitive impairment (n = 3, 1 male, 2 female), and dementia etiology unknown (n = 3, 1 male, 2 female). Additionally, CSF was obtained from healthy volunteers (n = 4, 2 male, 2 female).

All subjects were examined and CSF samples collected, divided into aliquots, and stored at -80°C until required. All assays were performed simultaneously to avoid repeated freezing and thawing of samples. The CSF samples were used within 1 month of collection.

We previously reported about 21 patients that suffered from early-stage CJD, defined as cases in the 6 weeks following the onset of the disease [6]. We used the CSF samples and clinical findings of these 21 patients. This study was approved by the Medical Ethics Committee of Nagasaki University, School of Medicine (06012755; UMIN000003301), and the participants provided written informed consent.

### 2. Analysis of CSF by WB for 14-3-3

The CSF samples were collected, aliquoted, and stored at -80°C until required. All assays were performed simultaneously to avoid repeated freezing and thawing of samples. Immunoassays for 14-3-3 protein were performed as previously described [3]. Polyclonal antibodies specific for 14-3-3γ were obtained from Immuno-Biological Laboratories (18647; Gunma, Japan) and were used at a dilution of 1:500. Polyclonal antibodies specific for all isoforms of 14-3-3 were obtained from Santa Cruz Biotechnology (sc-1657; Santa Cruz, CA, USA) and used at a dilution of 1:1, 000. All samples were analyzed using the same antibody to ensure comparable sensitivities. Protein detection was performed using an enhanced chemiluminescence detection kit (Amersham Buchler Company). Detection of 14-3-3 in the CSF samples was performed as previously described [3]. All assays were performed by two independent researchers.

### 3. Analysis of total tau protein in CSF samples

Detection of total tau protein in the CSF samples was performed as previously described [6].

### 4. ELISA detection of 14-3-3 in CSF

We immunized rabbits and mice with eight 14-3-3γ peptides and obtained three monoclonal antibodies (clones #4-#6; additional files 2, table S2) and three polyclonal antibodies (clones #1-#3; additional file 2, table S2). These six antibodies were characterized and the best combination of antibodies for the sandwich ELISA was determined (clones #1 and # 6; Additional file 3, figure S1). The sandwich ELISA for detection of 14-3-3γ was used to analyze CSF samples from 124 CJD patients and the remaining 99 patients with other diseases. The ELISA assay was sensitive enough to detect 14-3-3γ in the range 125-16, 000 AU/ml in human CSF samples. The ELISA plates were coated with antibody and then 50 μl of CSF and 50 μl of sample dilution buffer was added to each well. The samples were incubated for 1 hour at room temperature. Following incubation in the appropriate secondary antibody, samples were reacted with horseradish peroxidase and the optical density of each well at 450 nm was determined using a microplate reader. All processes were completed within a 4-hour period.

### 5. Analysis by the real-time QUIC (RT-QUIC) method

Analysis by the RT-QUIC method was performed as previously described [7].

### 6. Statistical analysis

SPSS version 11.0 software was used to perform all statistical analyses. Standard measures of diagnostic test validity were used to identify true-positive, true-negative, false-positive, and false-negative results. The levels of 14-3-3γ in the 124 CJD patients and remaining 99 patients with other diseases were used for these calculations [receiver operating characteristic (ROC) analysis].

### 7. MRI Protocol and PSWC on EEGs

We have described the MRI protocol and PSWC on EEGs previously [6].

## Results

### 1. Detection of 14-3-3 by WB in CSF samples

**Figure 1:**
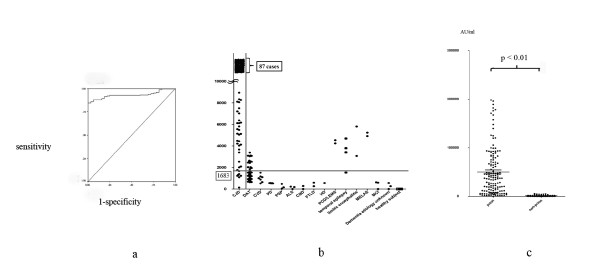
**ELISA analysis of 14-3-3 protein in CSF from patients with CJD and other neurological disorders**. 1-a. Receiver operating curve characteristics at different cut-off points for the 14-3-3 ELISA applied to CSF samples. 1-b. Results of the 14-3-3 ELISA analysis in CSF from patients with CJD and other forms of dementia. CJD, Creutzfeldt-Jakob disease; DAT, dementia of Alzheimer's type; CVD, cerebrovascular disorders; PD, Parkinson's disease; PSP, progressive supranuclear palsy; FTLD, frontotemporal lobular degeneration; HD, Huntington's disease; CBD, corticobasal degeneration; PCD/LEMS, paraneoplastic cerebellar disorder/Lambert-Eaton myasthenic syndrome; MELAS, mitochondrial myopathy, encephalopathy, lactic acidosis and stroke-like episodes; MCI, mild cognitive impairment. 1-c. Comparison of CJD patients and non-prion patients.

In the CJD group, 14-3-3 was detected in all CSF samples (n = 124; Tables 1 and 2). The 14-3-3 protein was detected in two non-CJD patients with DAT, one with CVD, two with Wernicke's encephalopathy, and three patients with limbic encephalitis. The WB sensitivity and specificity for 14-3-3γ in CJD patients was 87.1 and 84.8%, respectively (Tables 1 and 2, Figure 1-a, b, c). In other hand, WB sensitivity and specificity for all isoforms of 14-3-3 in CJD patients was 91.4 and 78.8% (Tables 1 and 2).

**Table 1:** The sensitivity and the specificity of ELISA of 14-3-3 **γ **protein, the detection of 14-3-3 **γ **protein and 14-3-3a protein in WB methods

	14-3-3 γ-isoform		14-3-3 all isoforms
	ELISA	WB	WB
sensitivity (%)	95.2	87.1	91.4
specificity (%)	72.7	84.8	78.8

**Table 2:** Analysis of Western blots method and ELISA of 14-3-3 protein of CSF in 124 CJD patients and 99 patients with other neurological disorders and rapid progressive dementia

disease	number	male	female	14-3-3 γ					14-3-3 all isoforms
				ELISA				WB	WB
				average	SD	min	max		
CJD	124	69	55	263, 549	21, 525	135	75, 373	108/124	114/124
DAT	54	33	21	1, 537	751.2	0	2, 410	3/54	7/54
CVD	7	5	2	975.9	332.4	521	1, 51 2	0/7	2/7
PD	5	4	1	542.2	20.3	521	565	0/5	0/5
PSP	3	2	1	223.3	226.6	38	476	0/3	0/3
FTLD	2	1	1	425	236.2	258	592	0/2	0/2
HD	1	1	0	556	0.	556	556	0/1	0/1
CBD	2	2	0	449.5	245.4	276	623	0/2	0/2
ALS	3	1	2	303.7	184.8	179	516	0/3	0/3
limbic encephalitis	2	1	1	4440.5	1935.4	3, 072	5, 809	2/2	2/2
MELAS	4	2	2	5, 069.5	222.7	4, 912	5, 227	4/4	4/4
PCD/LEMS	2	1	1	4, 387	217.8	4, 233	4, 541	2/2	2/2
temporal epilepsy	4	1	3	3, 356.8	1332.4	1, 530	4, 696	4/4	4/4
MCI	3	1	2	603	20.8	591	627	0/3	0/3
Dementia, etiology unknown	3	1	2	410.3	150.5	258	559	0/3	0/3
healthy subject	4	2	2	0	0	0	0	0/4	0/4

CJD: Creutzfeldt-Jakob disease, DAT: Dementia of Alzheimer's type, CVD: Cerebral Vascular Disorder, PD: Parkinson's disease, PSP: progressive supranuclear palsy, FTLD: frontotemporal lobular degeneration HD: Huntington's disease, CBD: corticobasal degeneration, ALS: amyotrophic lateral sclerosis, MELAS: Mitochondrial myopathy, Encephalopathy, Lactic Acidosis, Stroke-like episodes, PCD: paraneoplastic cerebellar degeneration, LEMS: Lambert-Eaton myasthenic syndrome, MCI: mild cognitive impairment,

### 2. Detection of total tau protein in CSF Samples

The levels of total tau protein in CSF were determined in 223 patients, and significant differences were observed among individuals (Tables 1 and 2). The level of total tau protein was highest in the CJD group, ranging from 1, 048-146, 087 pg/ml (mean ± SD, 7, 174 ± 6, 558 pg/ml). The CJD patients expressed higher levels of total tau proteins compared with patients suffering from other neurological disorders patient (*p *< 0.01; Tables 1 and 2, Figure 1).

### 3. Detection of 14-3-3 in CSF samples by ELISA

The levels of 14-3-3 in CSF were determined in 223 patients, with significant differences observed among individuals. The level of 14-3-3 was greatest in the CJD group, ranging from 135-75, 373 AU/ml (mean ± SD, 263, 549 ± 21, 525 AU/ml; Table 2-a). In the DAT group, the concentration of 14-3-3 was between 0-2, 410 AU/ml (mean ± SD, 1, 537 ± 751.2 AU/ml). The CVD group exhibited levels similar to those in the DAT group, with a range of 521-1, 512 AU/ml (mean ± SD, 975.9 ± 332.4 AU/ml). The level of 14-3-3 in CJD patients was also higher compared with patients suffering other neurological disorders, including the CVD and DAT groups (*p *< 0.01). A cut-off (1, 683 AU/ml) was determined by an ROC curve (Figure 1-a). The sensitivity and specificity for detection of 14-3-3 in CJD patients was 95.2 and 72.7%, respectively (Tables 1 and 2, Figure 1-a, b, c). We were able to demonstrate that these ELISA results were reproducible (additional file 4, figure S2-a, b)

### 4. Analysis of biochemical markers in 21 early-stage CJD patients

We previously published a report outlining 21 subjects that suffered from early-stage CJD, which was defined as cases in the 6 weeks following the onset of disease [6]. The sensitivities for detection of total tau protein and 14-3-3 in CSF were analyzed to determine their usefulness as diagnostic markers of early-stage CJD. Total tau and 14-3-3 proteins were detected in 95.2 and 76.2%, respectively, of the early-stage CJD patients. In all 21 patients, the data exceeded the cut-off data (> 1, 683 AU/ml) for the 14-3-3 ELISA (Table 3). Additionally, DWI-MRI was used to positively identify 90.5% of these cases. All cases were able to be diagnosed using a combination of DWI-MRI and 14-3-3γ ELISA.

**Table 3:** Summary of the detection of ELISA of 14-3-3 protein, the detection of WB method of 14-3-3 protein and in CSF for 21 patients with early-stage CJD

Age	Sex	CJD type		**d.w**.	CSF				MRI
						
					ELISA of 14-3-3 protein AU/ml	the detection of WB method of 14-3-3a protein	the detection of WB method of 14-3-3γ protein	Real-time QUIC	DWI
71	m	sp	probable	0	4, 157	-	-	+	+
77	m	sp	probable	2	10, 812	+	+	+	+
64	f	sp	probable	4	11, 812	+	+	+	+
73	m	sp	probable	4	3, 850	+	+	-	+
67	m	sp	probable	4	10, 814	+	+	+	+
76	m	sp	probable	4	6, 772	+	+	+	+
80	f	sp	probable	4	9, 850	+	+	+	+
63	f	sp	probable	4	2, 987	-	-	-	+
67	m	sp	probable	4	3, 553	+	+	+	+
70	f	sp	probable	4	3, 729	-	-	-	+
63	m	sp	probable	4	6, 133	+	+	+	+
63	m	sp	probable	4	8, 297	+	+	+	+
67	f	sp	probable	5	3, 372	+	-	-	-
74	m	sp	probable	5	7, 184	+	+	+	+
69	f	sp	definite	6	5, 600	+	+	+	+
54	f	sp	definite	6	1, 897	-	-	-	+
70	f	ia	probable	6	9, 460	+	+	+	-
70	f	fa	probable	6	4, 417	+	+	+	+
64	f	sp	probable	6	3, 888	+	+	+	+
51	f	ia	probable	6	6, 313	+	+	+	+
74	f	sp	probable	6	6, 133	+	+	+	+

sp = Sporadic CJD; ia = iatrogenic CJD; fa = familiar CJD; d.l. = diagnostic level based on the WHO and the Masters criteria; d.w. = duration from the onset of the disease to the diagnostic examination. In all 21 cases, codon 129 of PRNP was Met/Met homozygous, whereas codon 219 was Glu/Glu homozygous. Total protein contents of all the patients stayed within the normal range. All patients in this study were Asian.

### 5. Analysis of CJD patients where 14-3-3γ was not detected by WB yet the concentration of 14-3-3γ exceeded the ELISA cut-off level (Table 4)

In seven of the 124 CJD patients, 14-3-3γ was not detected by the WB but their values were above the cut-off level (1, 683 AU/ml) as determined by the 14-3-3γ ELISA (Table 4). The clinical courses of disease for the five sporadic CJD patients were longer when compared with the classical CJD patients.

**Table 4:** Analysis of CJD patient s that were not detected in 14-3-3γ protein of WB methods but were beyond the cut-off level (1, 683 AU/ml) in 14-3-3 protein ELISA.

Age	Sex	type	Polymorphism of codon129 in PRNP gene	Mutation of PRNP gene	ELISA of 14-3-3 protein (AU/ml)	Real-time QUIC
69	male	sp	MM	-	2, 101	+
33	female	sp	MM	-	1, 852	+
70	female	sp	MM	-	2, 452	-
73	female	sp	MM	-	2, 531	-
64	male	sp	MM	-	5, 493	+
65	male	fa	MM	E200K	4, 621	+
79	female	sp	VV	-	2, 342	+

sp: sporadic type, ge: genetic type, MM: methioine homozygotes, VV: valine homozygotes, +: positive, -: negative

### 6. Analysis of CJD patients where 14-3-3γ was not detected using WB and 14-3-3γ concentration was below the cut-off level for the ELISA (Table 5)

For seven of the 124 CJD subjects, 14-3-3γ was not detected using the WB method and the concentration of the protein was below the cut-off level (1, 683 AU/ml) as determined by the 14-3-3γ ELISA (Table 5). Five of the genetic CJD patients had the V180I mutation, and the remainder had the M232R slow-type mutation. The clinical courses of the disease in the five genetic CJD patients were longer than for the classical CJD patients.

**Table 5:** Analysis of CJD patient s that were not detected in 14-3-3γ protein of WB methods and were below the cut-off level (1, 683 AU/ml) in 14-3-3 protein ELISA.

Age	Sex	type	Polymorphism of codon129 in PRNP gene	Mutation of PRNP gene	ELISA of 14-3-3 protein (AU/ml)	Real-time QUIC
85	female	ge	MM	V180I	1, 086	-
84	female	ge	MM	V180I	1, 222	-
84	male	ge	MM	V180I	1, 313	-
84	female	ge	MM	V180I	1, 383	-
75	male	ge	MM	M232R	1, 676	+
78	male	sp	MM	-	135	-
66	male	sp	MM	-	1, 224	+

sp: sporadic type, ge: genetic type, MM: methioine homozygotes, VV: valine homozygotes, +: positive, -: negative

### 7. Analysis of non-CJD patients with positive 14-3-3γ ELISA diagnoses (Table 6)

The results from 23/99 of the non-CJD patients demonstrated that the concentration of 14-3-3γ exceeded the cut-off limit. When the concentration of 14-3-3γ was greater than 3, 000 AU/ml, the protein in the samples could also be detected by WB. Patients diagnosed with PCD/LEMS, MELAS, limbic encephalitis, temporal epilepsy, and a part of DAT were pseudo-positive, but negative using the RT-QUIC method.

## Discussion

We attempted to develop a sandwich ELISA using combinations of the specific γ-isoform antibodies (clones #1, #2 and #6: additional file 2, table S2), but only one combination of antibodies (#1 and #6: additional file 2, table S2) showed a dose-dependent reaction (Additional files 3, figure S1-a). The #1 and #6 antibodies reacted with the γ-isoform of 14-3-3.

There are clear advantages for using an ELISA over a WB for the detection of 14-3-3 in CSF samples. The WB method requires many steps for the detection of the target protein, with the WB protocol varying between laboratories. Thus, it is very difficult to obtain consistent data regarding the number, location and intensity of banding patterns. Moreover, the WB method can assay several samples simultaneously. It is an unsuitable method for patients in urgent need of treatment. However, the ELISA method allows for simultaneous processing of numerous CSF samples and appears to be the best method for use in a clinical setting.

In this study, we developed a sensitive and precise ELISA method for the quantification of 14-3-3 in CSF. The ELISA analysis of CSF in CJD patients was stratified by diagnosis category, and indicated a significantly higher 14-3-3 level in definite as well as probable CJD patients, compared to the CSF from patients with other neurological disorders.

Additionally, seven cases were analyzed by ELISA as the protein levels could not be determined by WB. These seven cases were classified into two categories (Tables 5 and 6). The first category consisted of samples with a total tau protein concentration exceeding 1, 300 pg/ml, with the concentration of 14-3-3 greater than 1, 683 AU/ml as determined by the ELISA. The second category consisted of samples with a total tau protein concentration less than 1, 300 pg/ml, but 14-3-3 concentration greater than 1, 683 AU/ml according to the ELISA (Table 5). All samples from CJD patients were assayed using the WB method for detecting 14-3-3 and the ELISA method for detecting total tau protein. This is because four patients exhibited a positive reaction for total tau (> 1, 300 pg/ml) and a negative reaction for 14-3-3 protein by WB; these four patients were also positive using the 14-3-3 ELISA (Table 4 and 5). We suggest that it might be unnecessary to check for both 14-3-3 and total tau proteins.

**Table 6:** The summary of non-CJD patients in the positive cases of ELISA of 14-3-3 **γ **protein

Age	Sex	diagnosis	WB (14-3-3 protein)		ELISA	Real-time QUIC method
			all isoforms	γ-specific isoform	γ-specific isoform	
79	male	PCD/LEMS	+	+	4, 233	-
69	male	PCD/LEMS	+	+	4, 541	-
51	female	temporal epilepsy	+	+	3, 405	-
59	female	temporal epilepsy	+	+	3, 796	-
62	male	limbic encephalitis	+	+	3, 072	-
78	male	limbic encephalitis	+	+	5, 809	-
42	female	MELAS	+	+	4, 912	-
25	female	MELAS	+	+	5, 227	-
78	male	DAT	+	+	3, 110	-
62	female	DAT	+	+	3, 386	-
69	female	DAT	+	+	3, 892	-
57	male	DAT	+	-	3, 092	-
59	male	DAT	+	-	3, 074	-
61	male	DAT	+	-	3, 038	-
66	female	DAT	+	-	2, 941	-
67	female	DAT	-	-	2, 569	-
70	female	DAT	-	-	2, 534	-
78	female	DAT	-	-	2, 445	-
83	female	DAT	-	-	2, 410	-
89	female	DAT	-	-	2, 025	-
72	male	DAT	-	-	1, 949	-
59	female	DAT	-	-	1, 852	-
63	male	DAT	-	-	1, 709	-

PCD: paraneoplastic cerebellar degeneration, LEMS: Lambert-Eaton myasthenic syndrome, MELAS: Mitochondrial myopathy, Encephalopathy, Lactic Acidosis, Stroke-like episodes, DAT: Dementia of Alzheimer's type

The present study varied from previous ELISA studies. In previous studies, all isoforms of 14-3-3 were detected, whereas in the present study we only detected the γ-isoform. The amount of 14-3-3γ was elevated in the CSF of CJD patients, consistent with previous WB studies. In a particular study [5], an ELISA method that measured all isoforms of 14-3-3 proved to be very useful. However, this system is not commercially available. Additionally, data from the ELISA method cannot be compared to previous results based on the β- or γ-isoform. However, the method described in the present study allows for these comparisons, with high specificity and sensitivity compared to other studies [2]. The differences in sensitivity and specificity for 14-3-3 diagnosis in various studies are likely due to the lack of uniformity between the diseases, as well as the number of diseases associated with 14-3-3. Previous studies [8,9] have shown that WB sensitivity and specificity for 14-3-3 are useful for diagnosis. However, the ELISA method is standardized, allowing for data comparison between subsequent studies. All ELISA procedures are completed within 4 hours, but the WB method requires 2-3 days. In addition, the ELISA assay can simultaneously analyze 40 samples from eight individuals.

Our previous report identified that the detection of total tau protein combined with DWI-MRI identified 98% of the early-stage cases. Pennington C et al [10] found that all three protein markers in CSF to be highly sensitive at the early stages of CJD, with CSF tau protein having the greatest specificity and efficiency. However, an ELISA to detect 14-3-3 was the most sensitive of the biochemical markers from the same samples. These findings indicate that a combination of DWI-MRI and 14-3-3γ ELISA are the most effective tests for detecting CSF protein markers during the early stages of CJD.

There were two problems with our study: 20 DAT cases identified that the level of ELISA was 3, 000-5, 000 AU/ml, but they could not be detected by WB and these results showed a discrepancy between the 14-3-3γ WB and ELISA (additional file 3, figure S1-a); and the specificity of the ELISA was very low (Figure 1). In this study we established an ELISA method for the detection of 14-3-3γ in CSF. The levels of 14-3-3 in the CSF of CJD patients were significantly elevated compared with those in other patients with neurological disorders. In various studies, the differences in sensitivity and specificity for detection of 14-3-3 were likely due to the lack of uniformity between the diseases, as well as the number of diseases that are associated with 14-3-3. The detection limit of the γ-isoform by WB was equivalent to 3, 400-4, 600 AU/ml by the ELISA, thus very low levels of 14-3-3γ in the CSF of DAT patients were confirmed using the ELISA (Table 6).

One of the reasons why the specificity was low was the combination of monoclonal antibodies used in the sandwich ELISA. If we had developed the ELISA using a combination of specific γ-isoform antibodies, such as clone #3, it is likely that the specificity may have been greater.

Atarashi et al. [7] detected the abnormal prion protein in CSF using the RT-QUIC method, with the specificity of the RT-QUIC method at 100%. These findings indicate the promise of an enhanced diagnostic capacity of RT-QUIC in the antemortem evaluation of suspected CJD. The RT-QUIC method has high specificity and moderate sensitivity in suspected CJD cases. Therefore, the cut-off limit for ELISA assay sensitivity was close to 100% because the specificity of the RT-QUIC method was 100%. Differential diagnosis is crucial, in particular because the number of false positive results may increase (Table 6). Additionally, the subtype of false negative results with human prion disease is also important (Tables 4 and 5).

## Conclusion

We have established an ELISA that specifically detects 14-3-3γ, and we believe that our ELISA is an appropriate primary diagnostic screening tool for human prion diseases. We think that the combination of an ELISA specific for 14-3-3γ and the RT-QUIC method are the best diagnostic tools that can be used to detect human prion diseases in CSF.

## Competing interests

Yuki Matsui, Katsuya Satoh, Toshiaki Miyazaki, Susumu Shirabe, Ryuichiro Atarashi, Kazuo Mutsukura, Yasufumi Kataoka and Noriyuki Nishida fulfill the criteria for Conflicts of Interest (COI) as determined by the conflict of interest committee of Nagasaki University and are protected by the "Policy of Conflict of Interest in Clinical Research".

All authors have no disclosures and no interest in the company, entity, or organization.

## Authors' contributions

YM and KM: YM and KM performed the analysis of the 14-3-3γ ELISA, WB and tau protein ELISA for human prion disease in CSF. TM: TM made the development of a 14-3-3γ ELISA kit and the charge nurse of the sale company of it SS: SS is a member of the CJD Surveillance Committee in Japan from 2007 till 2011. RA: RA performed the analysis of the RT-QUIC method for detecting human prion disease in CSF. AS and YK: investigators in this study. KS: KS analyzed the 14-3-3γ ELISA, Western blots and tau protein ELISA in CSF; KS is a member of the CJD Surveillance Committee in Japan on 2012; and chief investigator of this study. NN:NN was the chief manager of this study. All authors read and approved the final manuscript.

## Pre-publication history

The pre-publication history for this paper can be accessed here:

http://www.biomedcentral.com/1471-2377/11/120/prepub

## Supplementary Material

Additional file 1 **Table S1. The profiles of all researches in the sensitivity and specificity of ELISA kits of 14-3-3 protein in CJD patients**. ELISA: WB: Western blots method, N.E.: not exminated We compared the previous reports of ELISA kits of 14-3-3 protein in CSF of CJD patients with our data. Supplementary reference. 1. Geschwind MD, Martindale J, Miller D et al. Challenging the clinical utility of the 14-3-3 protein for the diagnosis of sporadic Creutzfeldt-Jakob disease. Archives of neurology. 2003;60:813-816. 2. Kenney K, Brechtel C, Takahashi H et al. An enzyme-linked immunosorbent assay to quantify 14-3-3 proteins in the cerebrospinal fluid of suspected Creutzfeldt-Jakob disease patients. Annals of Neurology. 2000; 48:395-398. 3. Gmitterova K, Heinemann U, Bodemer M et al. 14-3-3 CSF levels in sporadic Creutzfeldt-Jakob disease differ across molecular subtypes. Neurobiology of Aging. 2009; 30(11): 1842-50. Click here for file

Additional file 2 **Table S2. The characterization of three monoclonal antibodies and three polyclonal antibodies in six isoforms of 14-3-3 protein**. 14-3-3 proteins are a highly conserved family of multifunctional proteins which are primarily found in high levels in neurons. These proteins comprise seven distinct isoforms (β-isoform, γ-isoform, η-isoform, ε--isoform, ζ-isoform, τ-isoform and σ-isoform), but σ-isoform has not been detected in the human brain. We analyzed six isoforms (β-isoform, γ-isoform, η-isoform, ε--isoform, ζ-isoform and τ-isoform) in human CSF. We obtained the full-length gene encoding each isoform ((β-isoform, γ-isoform, η-isoform, ε--isoform, ζ-isoform and τ-isoform) of 14-3-3 protein from a cDNA library. Full-length constructs encoding either the β- or γ-isoform of human 14-3-3 protein in addition to a His-tag were cloned into pcDNA6/His vector, after which the constructs were transfected into murine 293T cell lines and over-expressed. All isoforms of protein were collected and purified three times through an affinity chromatography column. We analyzed all isoforms of recombinant protein of 14-3-3 protein reacted by three monoclonal antibodies (#1-#3) and polyclonal antibodies (#4-#6). Two monoclonal antibodies (#1 and #2) and one polyclonal antibody (#6) were specific by only γ-isoform of 14-3-3 protein. But one monoclonal antibody (#3) and two polyclonal antibodies (#4 and #5) were reacted by other isoforms including γ-isoform of 14-3-3 protein. Click here for file

Additional file 3 **Figures S1a and S1b. The standard curves obtained for the sandwich ELISA (clones #1 and #6)**. The standard curves obtained for the sandwich ELISA (clones #1 and #6). The standard control used a recombinant γ-isoform of 14-3-3. The combination of antibodies (#1 and #6) showed a dose-dependent reaction against the γ-isoform. **Detection of 14-3-3 by the Western blot method in DAT patients**. Detection of 14-3-3 by the Western blot method in DAT patients. Both cases 1 and 2 were DAT patients. The data from the 14-3-3 ELISA indicated that the protein concentration in cases 1 and 2 were 3, 168 and 6, 773 AU/ml, respectively. The positive control (#3) was also included. Click here for file

Additional file 4 **Figure S2a and S2b. The relationship between the concentration of standard samples and the absorbance**. The relationship between the concentration of standard samples and the absorbance. Correlation coefficient = 0.9967. **The relationship between the concentration of standard samples, and the absorbance in different standard samples**. The relationship between the concentration of standard samples, and the absorbance in different standard samples. These measurements were repeated five times. We acquired the almost data similar to the fifth. Click here for file
