# Supplementary material for: High sensitivity of an ELISA kit for detection of the gamma-isoform of 14-3-3 proteins: usefulness in laboratory diagnosis of human prion disease
Source: BMC Neurol. 2011 Oct 4;11:120. doi: 10.1186/1471-2377-11-120 (PMC3204235; doi:10.1186/1471-2377-11-120)
Supplement: Additional file 1 — Table S1. The profiles of all researches in the sensitivity and specificity of ELISA kits of 14-3-3 protein in CJD patients. ELISA: WB: Western blots method, N.E.: not exminated We compared the previous reports of ELISA kits of 14-3-3 protein in CSF of CJD patients with our data. Supplementary reference. 1. Geschwind MD, Martindale J, Miller D et al. Challenging the clinical utility of the 14-3-3 protein for the diagnosis of sporadic Creutzfeldt-Jakob disease. Archives of neurology. 2003;60:813-816. 2. Kenney K, Brechtel C, Takahashi H et al. An enzyme-linked immunosorbent assay to quantify 14-3-3 proteins in the cerebrospinal fluid of suspected Creutzfeldt-Jakob disease patients. Annals of Neurology. 2000; 48:395-398. 3. Gmitterova K, Heinemann U, Bodemer M et al. 14-3-3 CSF levels in sporadic Creutzfeldt-Jakob disease differ across molecular subtypes. Neurobiology of Aging. 2009; 30(11): 1842-50. [file 1471-2377-11-120-S1.DOC]

Additional files Table S1

Title: The profiles of all researches in the sensitivity and specificity of ELISA kits of 14-3-3 protein in CJD patients

|  | isoform | The number of patients (cases) | | Overall sensivity (%) | Specificity  (%) |
| --- | --- | --- | --- | --- | --- |
|  |  | prion disease | non-prion disease |  |  |
| Matsui et al | specific γ-isoform | 124 | 99 | 95.2 | 72.7 |
| Geschwind et al.^1^ | all isoforms | 32 | NE | 43 | N.E. |
| Kenney et al. ^2^ | all isoforms | 41 | 84 | 98 | 98 |
| Gmitterova et al.^3^ | all isoforms | 70 | 20 | 89 | 94 |

ELISA: WB: Western blots method, N.E.: not exminated

Description: We compared the previous reports of ELISA kits of 14-3-3 protein in CSF of CJD patients with our data.

Supplementary reference

1. Geschwind MD, Martindale J, Miller D et al. Challenging the clinical utility of the 14-3-3 protein for the diagnosis of sporadic Creutzfeldt-Jakob disease. Archives of neurology. 2003;60:813-816

2．Kenney K, Brechtel C, Takahashi H et al. An enzyme-linked immunosorbent assay to quantify 14-3-3 proteins in the cerebrospinal fluid of suspected Creutzfeldt-Jakob disease patients. Annals of Neurology. 2000; 48:395-398

3. Gmitterova K, Heinemann U, Bodemer M et al. 14-3-3 CSF levels in sporadic Creutzfeldt-Jakob disease differ across molecular subtypes. Neurobiology of Aging. 2009; 30(11): 1842-50
