# Supplementary material for: High sensitivity of an ELISA kit for detection of the gamma-isoform of 14-3-3 proteins: usefulness in laboratory diagnosis of human prion disease
Source: BMC Neurol. 2011 Oct 4;11:120. doi: 10.1186/1471-2377-11-120 (PMC3204235; doi:10.1186/1471-2377-11-120)
Supplement: Additional file 2 — Table S2. The characterization of three monoclonal antibodies and three polyclonal antibodies in six isoforms of 14-3-3 protein. 14-3-3 proteins are a highly conserved family of multifunctional proteins which are primarily found in high levels in neurons. These proteins comprise seven distinct isoforms (β-isoform, γ-isoform, η-isoform, ε--isoform, ζ-isoform, τ-isoform and σ-isoform), but σ-isoform has not been detected in the human brain. We analyzed six isoforms (β-isoform, γ-isoform, η-isoform, ε--isoform, ζ-isoform and τ-isoform) in human CSF. We obtained the full-length gene encoding each isoform ((β-isoform, γ-isoform, η-isoform, ε--isoform, ζ-isoform and τ-isoform) of 14-3-3 protein from a cDNA library. Full-length constructs encoding either the β- or γ-isoform of human 14-3-3 protein in addition to a His-tag were cloned into pcDNA6/His vector, after which the constructs were transfected into murine 293T cell lines and over-expressed. All isoforms of protein were collected and purified three times through an affinity chromatography column. We analyzed all isoforms of recombinant protein of 14-3-3 protein reacted by three monoclonal antibodies (#1-#3) and polyclonal antibodies (#4-#6). Two monoclonal antibodies (#1 and #2) and one polyclonal antibody (#6) were specific by only γ-isoform of 14-3-3 protein. But one monoclonal antibody (#3) and two polyclonal antibodies (#4 and #5) were reacted by other isoforms including γ-isoform of 14-3-3 protein. [file 1471-2377-11-120-S2.DOC]

Additional files Table S2

Title: The characterization of three monoclonal antibodies and three polyclonal antibodies in six isoforms of 14-3-3 protein

|  |  | β | γ | η | ε | ζ | τ |
| --- | --- | --- | --- | --- | --- | --- | --- |
| monoclonal antibodies | #1 | - | +++ | - | - | - | - |
|  | #2 | - | +++ | - | - | - | - |
|  | #3 | - | +++ | ++ | - | - | - |
| polyoclonal antibodies | #4 | ++ | ++ | ++ | + | ++ | ++ |
|  | #5 | ++ | + | - | - | + | - |
|  | #6 | - | +++ | - | - | - | - |

Description: 14-3-3 proteins are a highly conserved family of multifunctional proteins which are primarily found in high levels in neurons. These proteins comprise seven distinct isoforms (β-isoform, γ-isoform, η-isoform, ε—isoform, ζ-isoform, τ-isoform and σ-isoform), but σ-isoform has not been detected in the human brain. We analyzed six isoforms (β-isoform, γ-isoform, η-isoform, ε—isoform, ζ-isoform and τ-isoform) in human CSF. We obtained the full-length gene encoding each isoform ((β-isoform, γ-isoform, η-isoform, ε—isoform, ζ-isoform and τ-isoform) of 14-3-3 protein from a cDNA library. Full-length constructs encoding either the β- or γ-isoform of human 14-3-3 protein in addition to a His-tag were cloned into pcDNA6/His vector, after which the constructs were transfected into murine 293T cell lines and over-expressed. All isoforms of protein were collected and purified three times through an affinity chromatography column. We analyzed all isoforms of recombinant protein of 14-3-3 protein reacted by three monoclonal antibodies ( #1-#3) and polyclonal antibodies ( #4-#6). Two monoclonal antibodies ( #1 and #2) and one polyclonal antibody (#6) were specific by only γ-isoform of 14-3-3 protein. But one monoclonal antibody ( #3) and two polyclonal antibodies ( #4 and #5) were reacted by other isoforms including γ-isoform of 14-3-3 protein.
