# Supplementary figures and images for: High sensitivity of an ELISA kit for detection of the gamma-isoform of 14-3-3 proteins: usefulness in laboratory diagnosis of human prion disease
Source: BMC Neurol. 2011 Oct 4;11:120. doi: 10.1186/1471-2377-11-120 (PMC3204235; doi:10.1186/1471-2377-11-120)

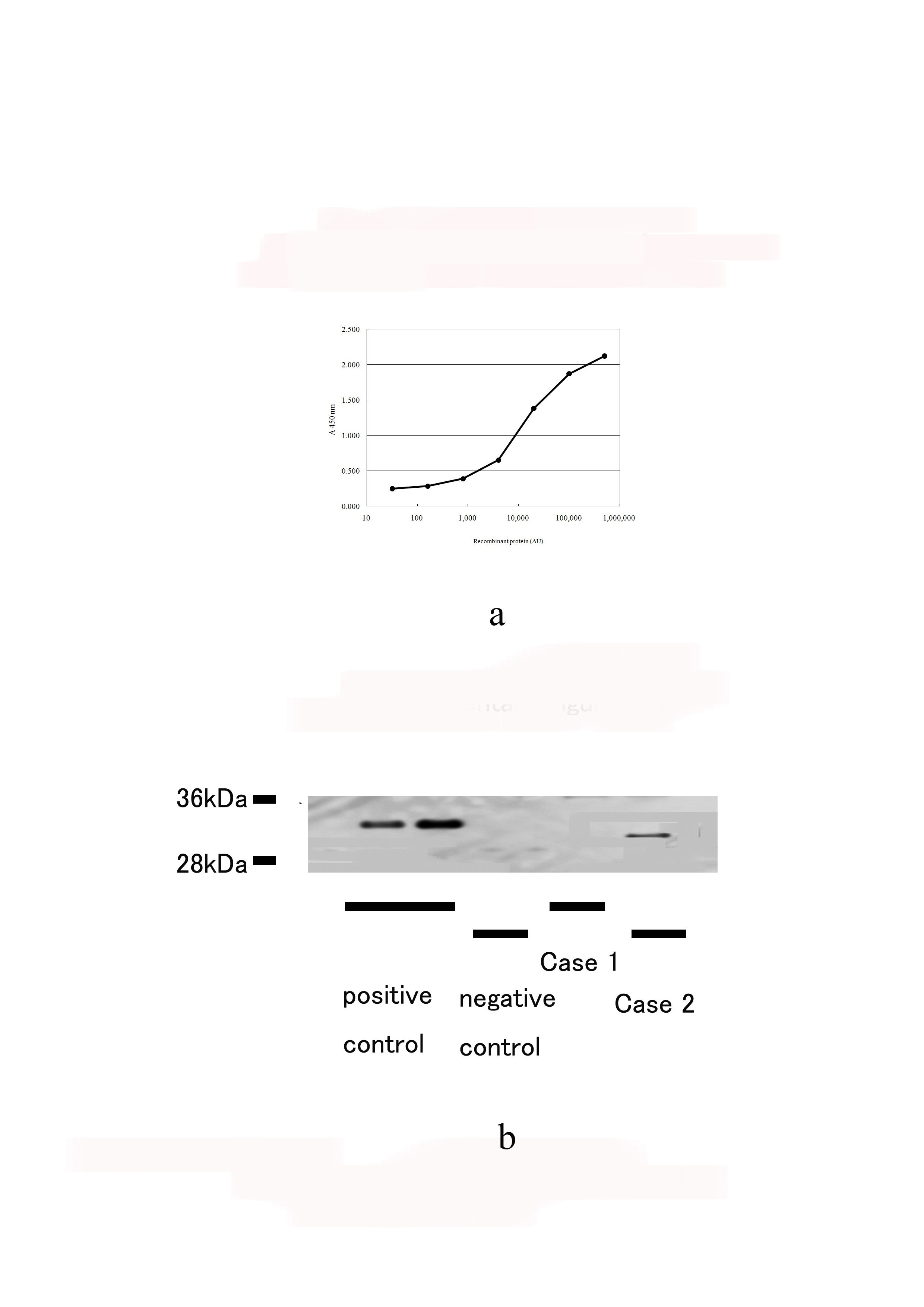

Supplement: Additional file 3 — Figures S1a and S1b. The standard curves obtained for the sandwich ELISA (clones #1 and #6). The standard curves obtained for the sandwich ELISA (clones #1 and #6). The standard control used a recombinant γ-isoform of 14-3-3. The combination of antibodies (#1 and #6) showed a dose-dependent reaction against the γ-isoform. Detection of 14-3-3 by the Western blot method in DAT patients. Detection of 14-3-3 by the Western blot method in DAT patients. Both cases 1 and 2 were DAT patients. The data from the 14-3-3 ELISA indicated that the protein concentration in cases 1 and 2 were 3, 168 and 6, 773 AU/ml, respectively. The positive control (#3) was also included. [file 1471-2377-11-120-S3.JPEG]

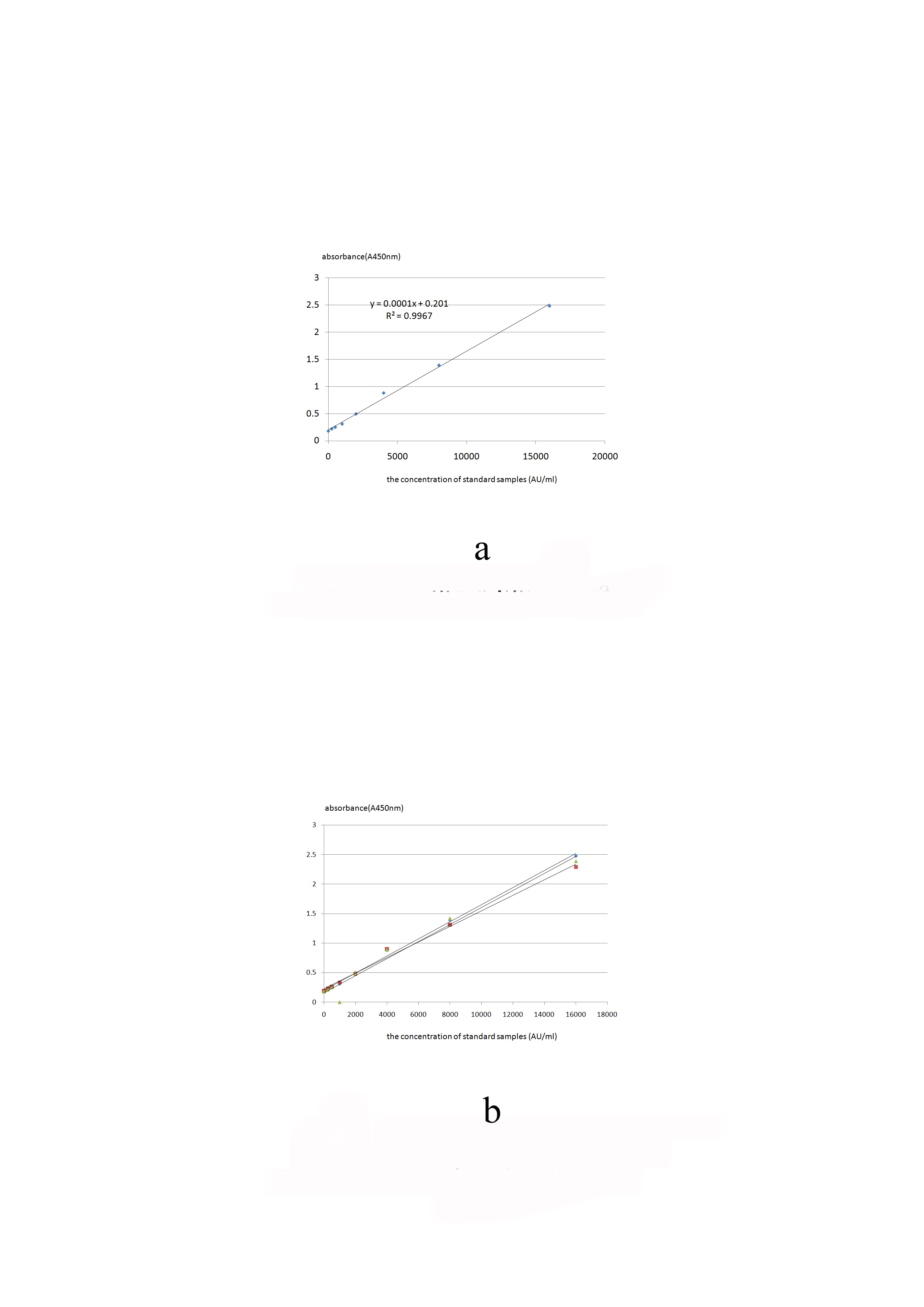

Supplement: Additional file 4 — Figure S2a and S2b. The relationship between the concentration of standard samples and the absorbance. The relationship between the concentration of standard samples and the absorbance. Correlation coefficient = 0.9967. The relationship between the concentration of standard samples, and the absorbance in different standard samples. The relationship between the concentration of standard samples, and the absorbance in different standard samples. These measurements were repeated five times. We acquired the almost data similar to the fifth. [file 1471-2377-11-120-S4.JPEG]
